# The effect of empagliflozin on peripheral microvascular dysfunction in patients with heart failure with preserved ejection fraction

**DOI:** 10.1186/s12933-025-02679-8

**Published:** 2025-04-25

**Authors:** Sanne G. J. Mourmans, Anouk Achten, Raquel Hermans, Marijne J. E. Scheepers, Elisa D’Alessandro, Geertje Swennen, Janneke Woudstra, Yolande Appelman, Harry van Goor, Casper Schalkwijk, Christian Knackstedt, Jerremy Weerts, Etto C. Eringa, Vanessa P. M. van Empel

**Affiliations:** 1 Department of Cardiology, Cardiovascular Research Institute Maastricht (CARIM), Maastricht University Medical Centre (MUMC+), Maastricht, The Netherlands; 2 Department of Physiology, Cardiovascular Research Institute Maastricht (CARIM), Maastricht, The Netherlands; 3 Department of Cardiology, Amsterdam UMC Heart Centre, Amsterdam, The Netherlands; 4 Department of Physiology, Amsterdam Cardiovascular Sciences, Amsterdam UMC, Amsterdam, The Netherlands; 5 Department of Pathology and Medical Biology, University of Groningen, University Medical Centre Groningen, Groningen, The Netherlands; 6 Department of Internal Medicine, Cardiovascular Research Institute Maastricht (CARIM), Maastricht University Medical Centre (MUMC+), Maastricht, The Netherlands

**Keywords:** Heart failure, Laser speckle contrast analysis, SGLT-2 inhibitor, Insulin, Acetylcholine, Nitroprusside, Endothelial function, Microcirculation

## Abstract

**Background:**

Empagliflozin is an effective treatment for heart failure with preserved ejection fraction (HFpEF), but its definite mechanism of action is unclear. Systemic microvascular dysfunction strongly relates to HFpEF aetiology, and we hypothesised that empagliflozin improves microvascular function in HFpEF.

**Objective:**

To investigate the effect of the sodium–glucose cotransporter-2 inhibitor empagliflozin on peripheral microvascular function in HFpEF.

**Methods:**

This is a pre-post intervention study in patients diagnosed with HFpEF who are eligible for treatment with empagliflozin. Microvascular function assessment using laser speckle contrast analysis of the dorsal forearm during iontophoresis of vasoactive stimuli (acetylcholine, insulin sodium nitroprusside) was performed at baseline and after 3 months of empagliflozin treatment (10 mg daily). The primary outcome was the difference in blood flow measured in the forearm microvasculature between baseline and at follow-up (cutaneous vascular conductance, CVC). Secondarily we investigated quality-of-life based on the EQ-5D-5 L questionnaire at baseline and follow-up.

**Results:**

Twenty six patients finished the study according to protocol (mean age of 74 ± 7 years, 62% female). We observed a decreased blood flow response to acetylcholine after 3 months of empagliflozin (CVC: 0.77 ± 0.24 vs. 0.64 ± 0.20, *p* < 0.001). In contrast, the response to insulin improved (CVC: 0.61 ± 0.43 vs. 0.81 ± 0.32, *p* = 0.03), and the response to sodium nitroprusside remained stable after 3 months. No significant correlations were found between the changes in blood flow and quality of life.

**Conclusion:**

This study shows that three months treatment with empagliflozin changed peripheral microvascular function in patients with HFpEF. Empagliflozin may enhance microvascular blood flow specifically via vascular actions of insulin, rather than a general effect on endothelial vasoregulation or smooth muscle cell function. As such, systemic microvascular dysfunction can be a modifiable factor in patients with HFpEF, while the clinical implications thereof warrant further investigations.

**Trial registration:**

The trial was preregistered at clinicaltrials.gov (NCT06046612).

**Graphical abstract:**

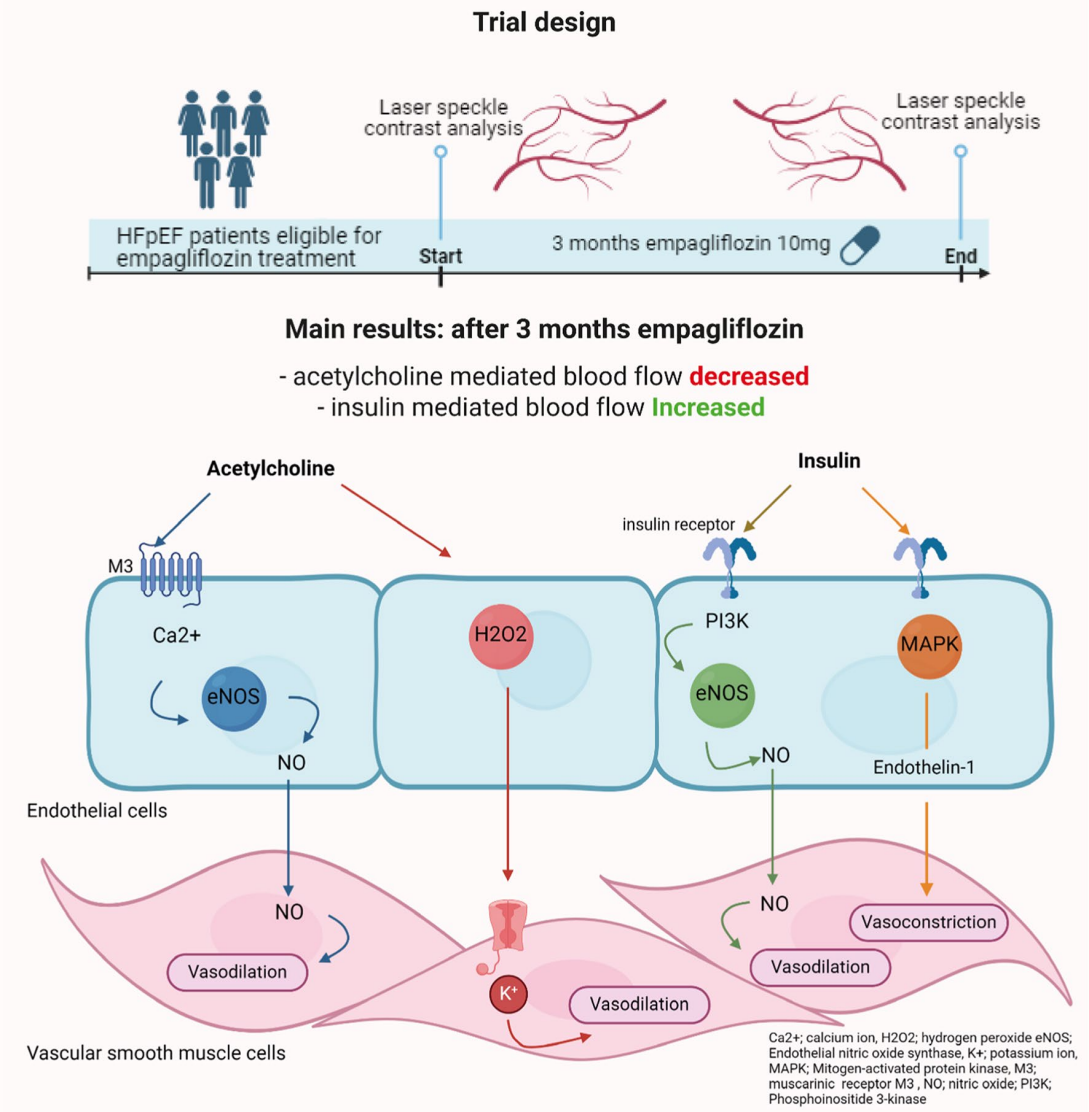

**Supplementary Information:**

The online version contains supplementary material available at 10.1186/s12933-025-02679-8.

## Research insights

**What is currently known about this topic?:**

SGLT-2 inhibitors are an effective treatment for patients with HFpEF, improving symptoms and outcomes. Systemic microvascular dysfunction is strongly implicated in the pathophysiology of HFpEF.

**What is the key research question?:**

Does SGLT-2 inhibitor empagliflozin improve peripheral microvascular function in patients with HFpEF?

**What is new?:**

This study identifies microvascular function as a modifiable factor in HFpEF. Empagliflozin enhances microvascular blood flow specifically via vascular actions of insulin, rather than a general effect on endothelial vasoregulation or smooth muscle cell function.

**How might this study influence clinical practice?:**

The findings can result in more specific treatments targeting endothelial dysfunction in HFpEF.

**Supplementary Information:**

The online version contains supplementary material available at 10.1186/s12933-025-02679-8.

## Introduction

Heart failure (HF) with preserved ejection fraction (HFpEF) is an important public health problem with a poor prognosis, as approximately half of the patients die within 5-years from their diagnosis. Its prevalence is largely driven by ageing and comorbidities such as diabetes, obesity and kidney disease. Systemic microvascular dysfunction (MVD) has been proposed to link these comorbidities to the development of HFpEF, by aggravating the deleterious effects of left ventricular (LV) diastolic dysfunction on peripheral organs [1–3]. Previous studies have consistently reported both coronary and peripheral MVD beds in patients with HFpEF [4–7]. Although the evidence is based on various techniques and vasoactive stimuli, it supports the concept that systemic MVD is a substrate for HFpEF [5, 6, 8, 9]. Also, systemic MVD has been associated with worsening of disease progression and cardiovascular outcomes in HFpEF [1, 10, 11].

The treatment options for patients with HFpEF remain limited. sodium–glucose cotransporter-2 (SGLT-2) inhibitors have been the first treatment for HFpEF proven to be beneficial. Empagliflozin reduced the combined risk of cardiovascular death or hospitalisation in patients with HFpEF in the EMPEROR-PRESERVED trial, independent of the glycaemic status [12]. Similar results were shown for dapaglifozin in the DELIVER trial in patients with HF and mildly reduced or preserved ejection fraction [13]. These drugs were initially recognised for their ability to improve glycaemic control in diabetes mellitus by increasing urinary glucose excretion due to blocking of the sodium–glucose co-transporter 2 in the proximal convoluted tubule of the nephron. This is particularly relevant, as patients with type 2 diabetes have a 25% lifetime risk of HF and their predominant subtype is HFpEF [14]. However, the EMPA-REG trial in diabetic patients with high cardiovascular risk showed that empagliflozin has pleiotropic effects, not only improving glycaemic control, but also renal and cardiovascular outcomes after a median follow-up of 3 years [15]. These effects, mediated by mechanisms such as glycosuria and natriuresis, may not fully explain the favourable cardiac effects of SGLT-2 inhibition. The cardio-protective effects of SGLT-2 inhibitors appear to be independent of improvements in the classical risk factors such as hypertension or hypercholesterolemia, because the benefits from these improvements usually require more time to become apparent [16–18].

Despite the absence of SGLT-2 receptors in the heart, empagliflozin may have direct cardiac effects. Preclinical studies have shown that SGLT-2 inhibitors might improve diastolic function in patients with HFpEF [19]. A study in non-diabetic mice demonstrated increased glucose and fatty acid oxidation, along with improved cardiomyocyte contractility [20]. However, clinical evidence of the effect of SGLT-2 inhibition on LV structure and function has been contradicting [21–23]. Furthermore, preclinical studies have suggested that empagliflozin may reduce inflammation and oxidative stress in HFpEF via multiple mechanisms [24, 25].

Understanding the effects of SGLT2 inhibition in HFpEF is essential to optimise its use in this growing population. However, no clinical research has been conducted to evaluate the effect of empagliflozin on systemic microvascular function in HFpEF. Based on previous evidence of systemic MVD in HFpEF, we hypothesised that empagliflozin improves systemic microvascular function in HFpEF. To allow more in-depth assessments of microvascular function modulation by empagliflozin in patients with HFpEF, we investigated peripheral microvascular function (changes in blood flow following vasodilation) through various pathways activated by different vasoactive stimuli.

## Methods

### Trial design

The current study is a pre-post intervention mono-centre study performed in MUMC+, the Netherlands. Patients with HFpEF who were eligible for treatment with empagliflozin but not yet using SGLT-2 inhibitors were identified by their treating physician. They underwent baseline peripheral microvascular function assessment before starting empagliflozin. All patients received 10 mg empagliflozin once daily for three months. After three months the assessment was repeated. Patients were instructed to inform the researcher if empagliflozin had to be discontinued. Therapy compliance was assessed verbally during the follow up visit. Participants were asked to complete a quality of life (QoL) questionnaire (Eq. 5D–5 L) during both visits. Based on previous preclinical and clinical research we considered three months to be sufficient to observe any significant differences in MV function [26–29]. The trial was preregistered at clinicaltrials.gov (NCT06046612). This study complies with the declaration of Helsinki and was approved by the Medical Ethics Committee University Hospital Maastricht/Maastricht University as a low-intervention clinical trial according to the Clinical Trial Regulation (EU CT 2022-501682-45). All patients provided written informed consent before enrolment.

### Study population

The current study was performed in patients diagnosed with HFpEF based on the 2021 ESC guidelines for HF at our specialized HFpEF outpatient clinic [30]. Patients underwent an elaborate workup for HFpEF diagnosis. Consensus on the diagnosis was reached between at least 2 HF cardiologists during a weekly meeting [31]. The study population consisted of newly diagnosed patients with HFpEF and patients visiting for their yearly check-ups.

Inclusion criteria were: HFpEF diagnosis, ability to understand and speak the Dutch language, treatment with empagliflozin 10 mg once daily planned to be started by the treating physician and written informed consent. Subjects were excluded from the study based on the following criteria: unable or unwilling to provide informed consent, under 18 years of age, contra-indication to the use of empagliflozin (severe kidney disease with glomerular filtration rate < 20 ml/min, severe liver insufficiency, recent or planned major surgery, severe acute disease such as acute coronary syndrome or stroke < 12 months, pregnancy), use of SGLT-2 inhibitor at baseline, known hypersensitivity to empagliflozin, acetylcholine, insulin, or sodium nitroprusside, insulin dependence in diabetes, enrolment or participation in another investigational device or drug study within the past 30 days, any condition that interferes with the correct execution of the laser speckle contrast analysis measurements (such as inability to keep arms motionless during the measurements, any condition that does not allow disposables to be attached to the forearm skin), or any other reason that makes it undesirable for patient to use empagliflozin according to the researcher or treating physician.

### Study procedures/assessment of peripheral microvascular function

Peripheral microvascular function was assessed using laser speckle contrast analysis (LASCA). LASCA enables non-invasive and continuous monitoring of microvascular blood flow in superficial microvascular beds, such as those in the skin. LASCA allows the evaluation of microvascular responses to different vasoactive agents administered by iontophoresis. Iontophoresis is a non-invasive method of transdermal drug delivery based on the transfer of charged ions/molecules due to a low-intensity electric current. The electrical circuit consisted of a drug delivery electrode with a 1.54 cm^2^ chamber to hold liquid, and a dispersive electrode placed at 10–15 cm distance from the latter. This procedure includes iontophoresis of acetylcholine (Ach), insulin (INS) and sodium nitroprusside (SNP). This combination of stimuli allows the evaluation of endothelium-dependent (Ach and INS) and endothelium-independent (SNP) microvascular function. These aspects of microvascular function have different regulating mechanisms in physiology and pathophysiology, resulting in a need for multiple stimuli [32–35]. While Ach and INS both stimulate nitric oxide (NO) production, INS additionally activates the endothelin-1 (ET-1) pathway providing complementary insights into endothelial dependent microvascular function [36, 37]. SNP acts as a direct NO donor, bypassing the endothelium to cause smooth muscle cell relaxation [34].

To improve reproducibility of the measurements, the following circumstances were taken into account: Measurements were started after a 30-min acclimation period in a temperature-controlled (22–23 °C) room to avoid confounding effects of temperature. No exercise was allowed before measurements and measurements were performed in the fasting state (medication use allowed), to minimise variation of blood flow due to physical activity and endogenous insulin production. A standardised protocol was used to perform LASCA and iontophoresis (Appendix 1).

### Primary and secondary endpoints of microvascular function

**Fig. 1:**
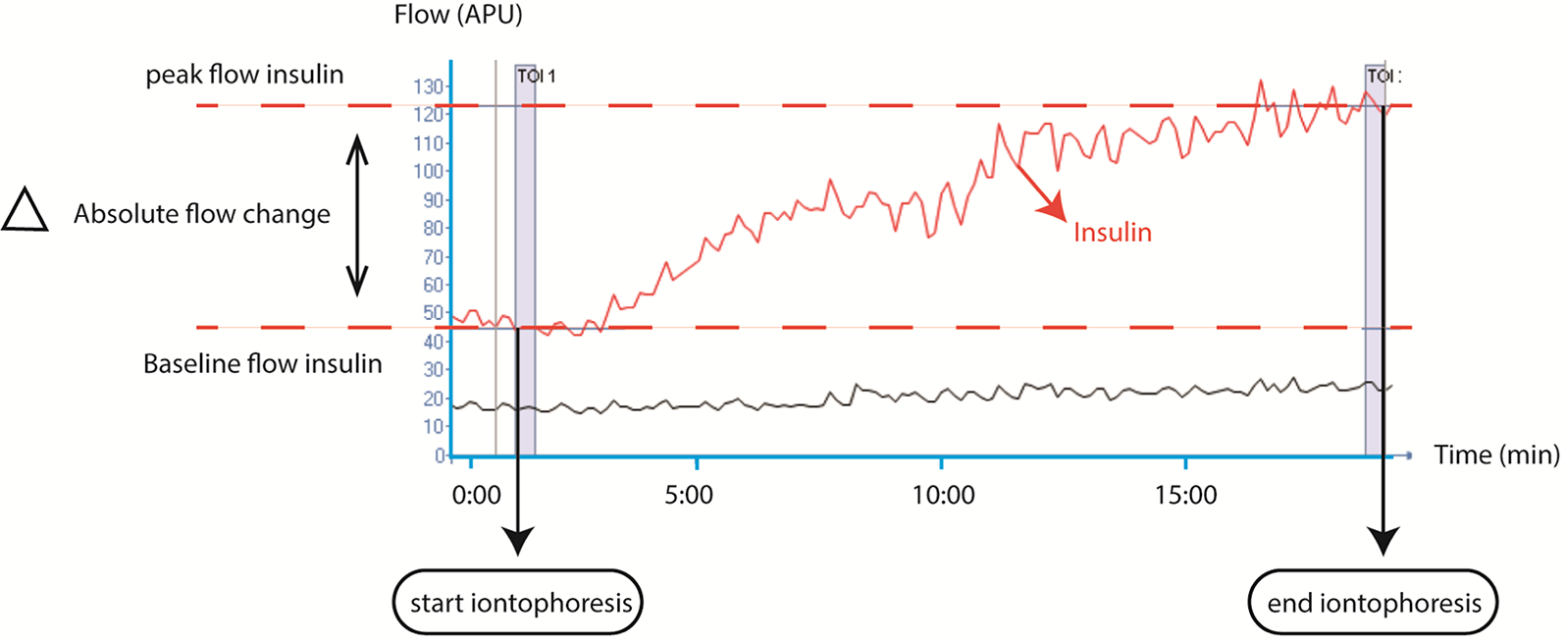
Example measurement for laser speckle contrast analysis during iontophoresis of insulin

The primary endpoint was the change in cutaneous vascular conductance (CVC) in response to acetylcholine, insulin, and sodium nitroprusside between baseline and after 3 months of empagliflozin treatment. CVC is the ratio between the blood flow increase during iontophoresis of the vasodilator (peak–baseline), and the mean arterial pressure (Fig. 1), calculated by the formula CVC = (Q_peak_−Q_baseline_)/MAP where Q is the cutaneous blood flow.

Blood flow was expressed in arbitrary perfusion units (APU) and mean arterial pressure in mmHg. CVC was measured at the baseline and 3-month follow-up visits. Secondary endpoints were peak blood flow (APU), absolute change in blood flow (APU), and area under the curve (AUC) (Fig. 1) LASCA data was obtained using dedicated software (Pimsoft version 1.11).

The X-axis represents time in minutes, the Y-axis represents blood flow in arbitrary perfusion units (APU). The baseline flow is the average flow (APU) during the first 30 s of the protocol. The peak flow is the average flow (APU) during the 30 s of highest blood flow. The absolute flow change is the delta between peak and baseline flow (APU).

### Quality of life

Quality of life was assessed using the EQ-5D-5 L questionnaire at baseline and after 3-months of follow-up. The data were collected through physical copies of the questionnaires during these two study visits. Participants were instructed to report their QOL as they felt on that particular day. The EQ-5D-5 L evaluates five dimensions: mobility, self-care, daily activities, pain/discomfort, and anxiety/depression, which are described in detail elsewhere [38]. The EQ-5D index score was derived from patients’ responses using the Dutch value set [39]. The visual analogue scale (VAS) was obtained to assess patients’ self-rated overall health status. The VAS can range from 0 (“worst health you can imagine”) to 100 (“best health you can imagine”). In addition, The Paretian Classification of Health Change (PCHC) was used to compare individual changes from baseline to 3-month follow-up [40].

### Measurement of blood markers of metabolism and vascular function

In all patients we determined blood markers of vasoreactivity (endothelin 1), insulin sensitivity (fasting insulin), inflammation (C-reactive protein) and oxidative stress (free thiols). Adiponectin was measured in plasma as a potential determinant of microvascular insulin responses. Serum and plasma samples were obtained from all patients in the fasting state at baseline and after 3 months of follow-up. The samples were centrifuged for 10 min (2000 g-max) and frozen at − 80 degrees Celsius until use.

Insulin, triglycerides and C-reactive protein were determined in serum using standard assays.

An endothelin ELISA kit (R&D systems, catalog number DET100, USA) was used to determine endothelin-1 (ET-1) levels in EDTA plasma. ET-1 levels were measured in duplicate. We assessed whether empagliflozin reduces plasma ET-1, and whether the effect of empagliflozin on plasma ET-1 relates to its effect on insulin-stimulated blood flow in skin. Insulin-stimulated blood flow in skin may be a correlate of ET-1 bioactivity, and therefore may relate to ET-1 levels in plasma. Free plasma thiols, a biomarker for systemic oxidative stress, were measured as previously described [41] and expressed relative to the concentration of albumin. Adiponectin was measured with the Human Adiponectin Kit of Meso Scale Discovery^®^ (Rockville, MD, US) with intra-assay variation coefficient (VC) of 2.4% and inter-assay VC of 2.8%.

### Sample size calculation

A sample size calculation was performed based on the Hoorn diabetes Care cohort [42], assuming a mean change in Ach-induced CVC + 0.16 APU/mmHg. The Ach-induced CVC response was selected as the primary measure for this calculation, due to its endothelium-dependent effects, which are relevant to HFpEF pathophysiology. Additionally, it is a more widely established stimulus for evaluating microvascular function in cardiovascular research, compared to INS. The mean change was based on a baseline mean of 0.99 APU/mmHg, and a standard deviation of the change of 0.25 APU/mmHg, and a within-person correlation of 0.8. Sample size calculation using G-Power (with a 2-sided level of significance α = 0.05, power 1−β = 0.80, and effect size 0.440) yielded a required sample size of *n* = 43. To compensate for possible drop-out, we increased the aimed total study population size by 10% to 48.

### Interim analysis

As per protocol, an interim analysis was performed after 50% of the study participants (*n* = 24) completed the full study, to reassess the required sample size. The found mean change in CVC for acetylcholine was − 0.11 with an SD of 0.15. The sample size calculation using G-power (with a 2-sided level of significance α = 0.05, power 1-β = 0.80, and effect size − 0.733) yielded a required sample size of *n* = 13. Based on these results, the study was closed for new inclusions, and was ended after all planned follow-up visits had taken place.

### Statistical analysis

A statistical plan was created prior start of study inclusions and is summarised here. Continuous baseline characteristics are described as mean ± SD, or median and first and third quartile in case of skewed distribution. Categorical characteristics are described as count and percentage. Baseline characteristics between patients in the per protocol group (*n* = 26) and the patients who are only included in the intention to treat group (*n* = 11) were tested using Pearson’s chi-square test or Fisher’s exact test as appropriate for categorical variables and the paired-samples *t*-test or Mann-Whitney *U*-test for continuous variables. Pearson’s correlation was used to identify correlations between variables.

All outcome parameters are compared between baseline and follow-up after 3 months. Data was analysed using the paired samples *t*-test or Wilcoxon signed-rank test as appropriate. A sensitivity analysis for the primary endpoint was performed using the intention-to-treat data rather than the per-protocol data.

Clinical correlates for the change in CVC in response to Ach, INS and SNP between baseline and after 3 months of follow-up were analysed using univariable and multivariable linear regression. Acknowledging the sample size, a multivariable model was prespecified to contain age, sex, and NYHA class.

For the QOL analysis summary statistics were derived, including the number of patients and proportions of categorical responses for the five EQ-5D dimensions. Correlation between the CVC and both EQ-5D index and visual analogue scale (VAS) were calculated.

The relation between the effects of empagliflozin on plasma ET-1 and adiponectin levels and microvascular function in the forearm skin was assessed using linear regression analysis. When normality testing of data revealed a lognormal distribution, data were log-transformed before analyses.

## Results

### Patient data availability

**Fig. 2:**
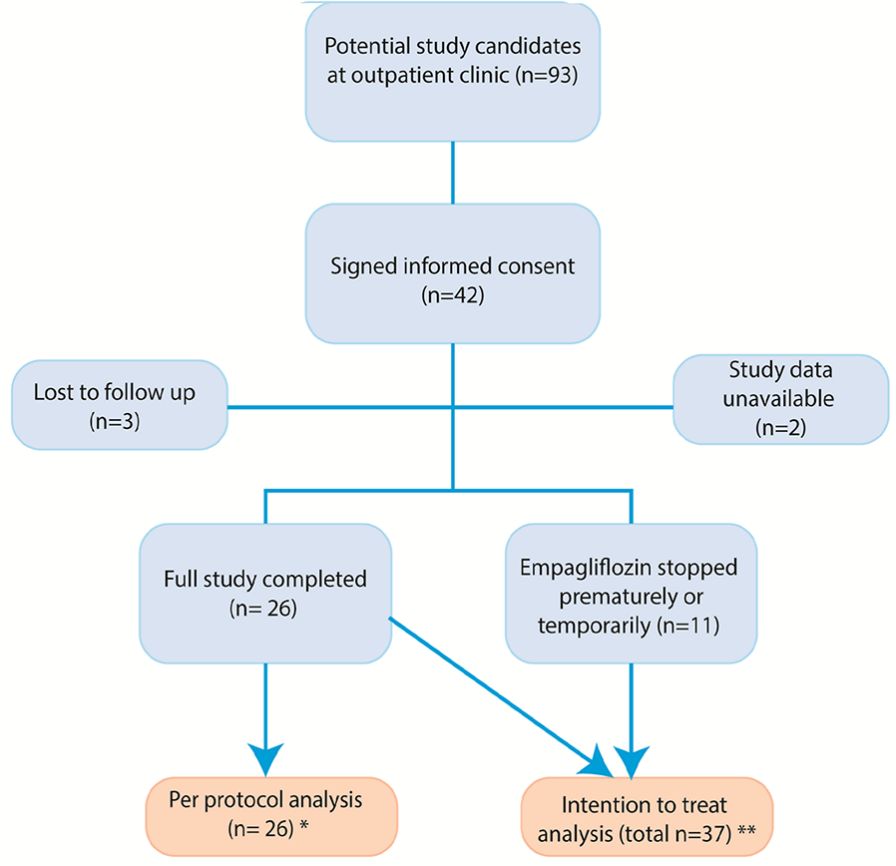
Study flow diagram. *Applicable to acetylcholine and sodium nitroprusside analyses. *N* = 25 for insulin per protocol analysis, due to 1 non-valid measurement. ** Applicable to acetylcholine and sodium nitroprusside analyses. *N* = 35 for insulin intention to treat analysis, due to 2 non-valid measurements

From February 2023 until February 2024 in total 42 patients were enrolled in this study. Three patients were lost to follow-up, as they were not able to attend the follow up visit due to events unrelated to study medication or study procedures. LASCA data were unavailable for two patients due to software malfunction. These two patients were excluded from the primary analysis but remained in the QoL analysis. Insulin data were missing for two patients, because one LASCA measurement was stopped prematurely on the patient’s request, and one failed due to a technical issue with insulin iontophoresis. The final analysis included 26 patients who completed the study according to protocol (per protocol analysis). These 26 patients took empagliflozin daily for 3 months and had complete primary endpoints available. The remaining eleven patients stopped empagliflozin prematurely or missed doses during the three month follow-up, with a median of 36 missed doses [7–65]. The most common reasons for non-adherence were side effects or forgetting to take the medication. These 11 patients were included in the intention-to-treat analysis. Detailed numbers per group are shown in Fig. 2.

### Population characteristics

Baseline characteristics are shown in Table 1. The 26 patients included in the final analysis had a mean age of 74 ± 7 years, and 62% were female. The most prevalent comorbidities were hypertension and atrial fibrillation. Only one patient had diabetes mellitus. Half of all patients were using loop diuretics and beta blockers at baseline. The baseline characteristics of the patients in the final analysis (*n* = 26) were comparable to the 11 patients that prematurely stopped empagliflozin or missed doses.

**Table 1:** Baseline clinical characteristics

Characteristics	Per protocol (*n* = 26)	Intention to treat (*n* = 37)
Age (years)	74 ± 7	74 ± 7
Female	16 (62%)	22 (60%)
Body mass index (kg/m^2^)	29 [26–36]	30 [26–36]
Mean arterial pressure (mmHg)	101 ± 13	102 ± 13
Systolic BP (mmHg)	149 ± 24	147 ± 22
Diastolic BP (mmHg)	78 ± 10	79 ± 11
Heart rate (/min)	62 ± 13	62 ± 13
*NYHA functional class*		
I	3 (12%)	3 (8%)
II	19 (73%)	28 (76%)
III	4 (15%)	6 (16%)
IV	0	0
*Medication*		
Loop diuretics	14 (54%)	20 (54%)
Thiazide diuretics	3 (12%)	4 (11%)
Beta blockers	14 (54%)	22 (60%)
ACE inhibitors	11 (42%)	12 (35%)
Angiotensin receptor blockers	5 (19%)	8 (22%)
Calcium antagonists	11 (42%)	14 (38%)
Long-acting nitrates	2 (8%)	2 (5%)
*Comorbidities*		
Significant CAD^b^	7 (27%)	8 (22%)
Atrial fibrillation	15 (58%)	19 (51%)
Hypertension	19 (73%)	26 (70%)
Diabetes mellitus	1 (4%)	3 (8%)
Hypercholesterolemia	8 (31%)	15 (41%)
Kidney disease^c^	12 (46%)	14 (38%)
Iron deficiency	8 (31%)	13 (35%)
COPD	4 (15%)	7 (19%)
Asthma	5 (19%)	5 (14%)
Current smoking	1 (4%)	1 (3%)
Stroke	1 (4%)	1 (3%)
Sleep apnoea	11 (42%)	18 (49%)
*Echocardiography*		
LV ejection fraction (%)	60 ± 5	60 ± 5
Interventricular septum (mm)	9 [9–11]	9 [8–11]
LV posterior wall (mm)	9 [ 8–10]	9 [8–10]
LV mass index (gr/m^2^)	86 ± 18	83 ± 17
Relative wall thickness	0.36 [0.34–0.41]	0.36 [0.34–0.43]
LA volume index (ml/m^2^)	44 [38–54]	43 [37–52]
E/A ratio	0.9 [0.7–1.6] (*n* = 15)	0.9 [0.7–1.6] (*n* = 23)
Septal e’ (cm/s)	6.7 ± 1.8 (*n* = 25)	6.6 ± 1.8 (*n* = 35)
Lateral e’ (cm/s)	9.1 ± 3.0 (*n* = 24)	8.9 ± 2.8 (*n* = 34)
Septal E/e’	12.9 [11.3–17.9] (*n* = 23)	13.4 [11.3–18.4 ] (*n* = 32)
Lateral E/e’	10.2 [8.5–12.8] (*n* = 22)	10.4 [8.6–12.8 ] (*n* = 31)
E/e’average	11.6 [ 9.3–14.3] (*n* = 23)	11.7 [9.3–14.9] (*n* = 32)
TR peak velocity (m/s)	2.6 ± 0.3 (*n* = 21)	2.6 ± 0.3 (*n* = 32)
*Laboratory values*		
NT-proBNP (pmol/l)	41 [28–109]	43 [29–100]
Creatinine (umol/l)	92 [77–111]	90 [ 79–103]
eGFR (mL/min/1.73m^2^)	60 ± 15	62 ± 15
Haemoglobin (mmol/l)	8.3 ± 0.9	8.4 ± 1.0
Hba1c (mmol/mol)	38 [36–41] (*n* = 25)	39 [35–41] (*n* = 35)

Data presented as count (percentage), median [interquartile range], or mean ± standard deviation, as appropriate. BP, blood pressure; CAD, coronary artery disease; COPD, chronic obstructive pulmonary disease; eGFR (CKD-EPI), estimated glomerular filtration rate according to the Chronic Kidney Disease Epidemiology Collaboration equation; LA, left atrium; LV, left ventricle; NT-proBNP, N-terminal pro B-type natriuretic peptide; NYHA, New York Heart Association; TR, tricuspid regurgitation

^b^significant CAD was defined as stenosis > 70%

^c^kidney disease was defined as glomerular filtration rate < 60 mL/min/1.73m^2^ during more than 3 months (at least 2 or more measurements)

### Laser speckle contrast analysis—per protocol analysis

**Fig. 3:**
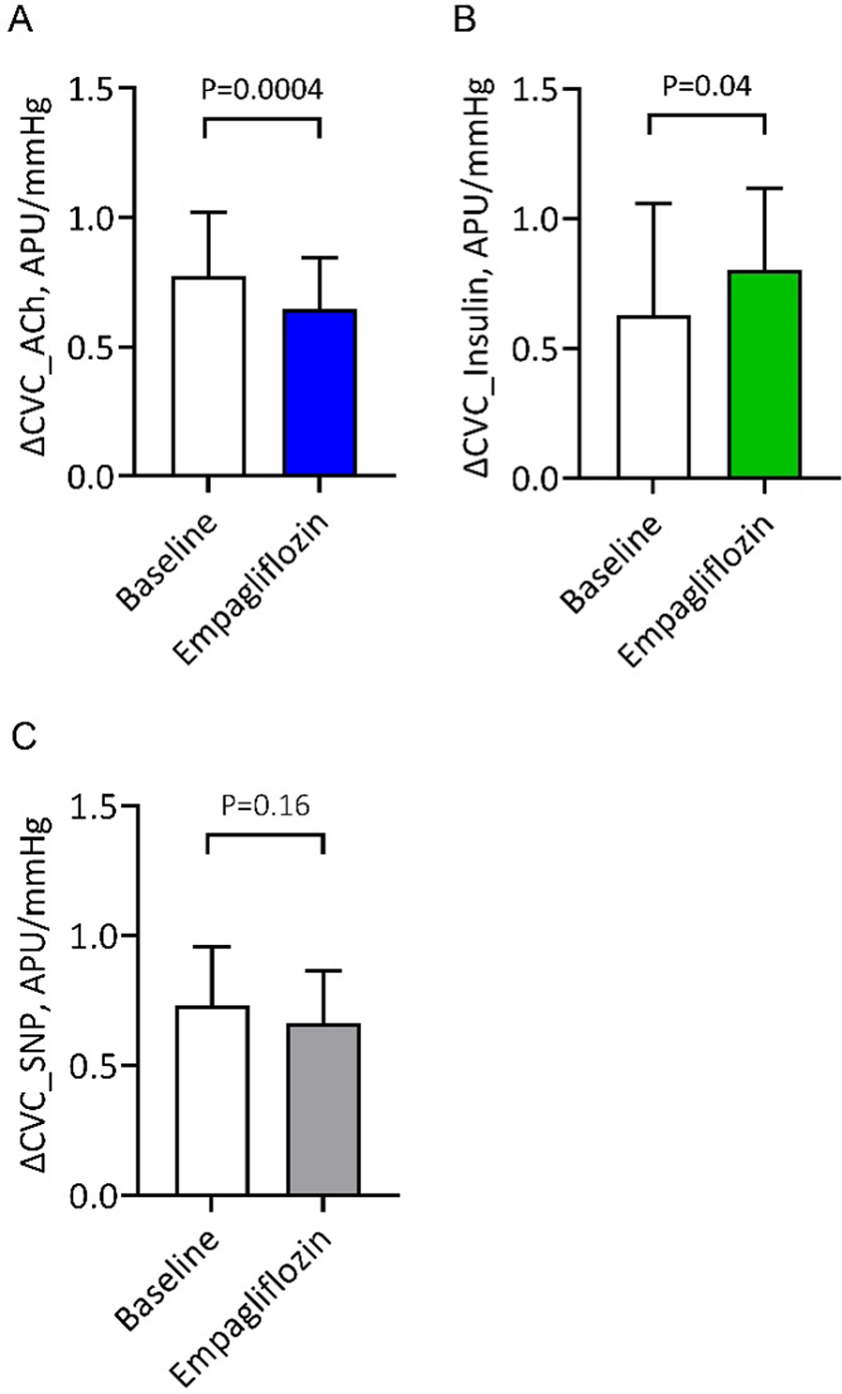
Changes in cutaneous vascular conductance at baseline and after 3 months of empagliflozin treatment. The results are shown during iontophoresis of acetylcholine (**A**), insulin (**B**) and sodium nitroprusside (SNP; **C**)

When comparing microvascular responses between baseline and follow-up after 3 months of empagliflozin, we observed a significant decrease in CVC during iontophoresis of acetylcholine (0.77 ± 0.24 vs. 0.64 ± 0.20, *p* < 0.001) (Table 2; Fig. 3). BMI was a univariable significant predictor of this CVC response, although it lost significance after adjusting for age, sex, and NYHA class (Table 3). Conversely, a significant increase of CVC in response to insulin iontophoresis was observed after 3 months of empagliflozin (0.62 ± 0.43 vs. 0.82 ± 0.31, *p* = 0.040) (Table 2; Fig. 3). Left atrial volume index (LAVI) was a univariable significant predictor of this CVC response and remained significant after adjusting for age, sex, and NYHA class (Table 3).

Age, sex, and NYHA class were not significant univariable predictors of CVC response to either acetylcholine or insulin. The effect of SNP on CVC did not significantly change after 3 months of empagliflozin treatment, but showed a decreasing trend (0.75 ± 0.23 vs. 0.65 ± 0.21, *p* = 0.086) (Table 2; Fig. 3). Creatinine values were predictors of this CVC response and remained significant after adjusting for age, sex, and NYHA class (Supplemental table S1).

**Table 2:** Blood perfusion measured by laser speckle contrast analysis during iontophoresis of acetylcholine, insulin, and sodium Nitroprusside

	Baseline	Follow-up at 3 months	*P*-value
*Acetylcholine (n = 26)*			
Baseline perfusion (APU)	51 [43–59]	42 [36–49]	0.005
Baseline perfusion (APU/mmHg)*	0.51 ± 0.12	0.46 ± 0.13	0.064
Peak perfusion (APU)	130 ± 30	107 ± 21	< 0.001
Peak perfusion (APU/mmHg)*	1.29 ± 0.27	1.10 ± 0.24	< 0.001
Absolute change (APU)	78 ± 27	62 ± 19	< 0.001
CVC (APU/mmHg)*	0.77 ± 0.24	0.64 ± 0.20	< 0.001
Area under the curve	23,520 [17583–29577]	16,841 [11594–24943]	0.011
*Insulin (n = 25)*			
Baseline perfusion (APU)	44 ± 10	44 ± 12	0.987
Baseline perfusion (APU/mmHg)*	0.44 ± 0.12	0.45 ± 0.13	0.719
Peak perfusion (APU)	106 ± 39	123 ± 29	0.054
Peak perfusion (APU/mmHg)*	1.07 ± 0.42	1.27 ± 0.33	0.044
Absolute change (APU)	62 ± 42	79 ± 29	0.051
CVC (APU/mmHg)*	0.62 ± 0.43	0.82 ± 0.31	0.040
Area under the curve	32,400 [7365–67016]	49,336 [37593–66808]	0.032
*Sodium nitroprusside (n = 26)*			
Baseline perfusion (APU)	56 ± 16	45 ± 11	0.002
Baseline perfusion (APU/mmHg)*	0.56 ± 0.16	0.47 ± 0.14	0.008
Peak perfusion (APU)	136 [115–152]	106 [96–123]	0.004
Peak perfusion (APU/mmHg)*	1.31 ± 0.27	1.12 ± 0.21	0.008
Absolute change (APU)	75 ± 23	64 ± 22	0.051
CVC (APU/mmHg)*	0.75 ± 0.23	0.65 ± 0.21	0.086
Area under the curve	37,026 [27180–46277]	31,730 [20793–39098]	0.028
*Metabolic parameters (n = 26)*			
Weight (kg)	82 [75–96]	81 [71–97]	0.038
BMI ( kg/m2)	29 [26–35]	28 [26–35]	0.091
Mean arterial pressure (mmHg)	101 ± 13	99 ± 14	0.24
Systolic blood pressure (mmHg)	149 ± 24	142 ± 21	0.10
Diastolic blood pressure (mmHg)	78 ± 10	77 ± 11	0.65
Fasting triglycerides (mM)	1.2 (1.0-1.7)	1.4 (1.0-1.6)	0.30
Fasting insulin (pmol/L)	73 (59–116)	70 (46–123)	0.14
C-reactive protein (mg/L)	2.6 (0.8-6.0)	2.2 (1.2–5.7)	0.57
Free thiols (umol/g albumin)	8.9 ± 0.8	8.8 ± 1.0	0.35

Data presented as median [interquartile range], or mean ± standard deviation, as appropriate. *APU* Arbitrary perfusion units, *CVC* Cutaneous vascular conductance (Absolute change/mean arterial pressure). *perfusion/mean arterial pressure

**Table 3:** Predictors for differences in blood flow response between baseline and 3 months follow up

	Unadjusted			Adjusted *		
	Beta (95% CI)	Standardised beta	*p*-value	Beta (95% CI)	Standardised beta	*p*-value
*Acetylcholine*						
BMI	− 0.014 (− 0.025 to − 0.02)	− 0.439	0.025	− 0.011 (− 0.025 to 0.003)	− 0.363	0.114
*Insulin*						
LAVI	0.017 (0.003–0.032)	0.468	0.017	0.019 ( 0.002 to 0.035)	0.502	0.027

*BMI* Body mass index, *LAVI* Left atrial volume index

*Adjusted for age, sex, and New York Heart association class

### Laser speckle contrast analysis—intention to treat analysis

In the intention to treat population, including patients who missed doses of empagliflozin during the 3 months follow-up (*n* = 37 for Ach and SNP, *n* = 35 for INS), we did not observe significant changes in CVC or AUC after 3 months in response to iontophoresis of acetylcholine, insulin, sodium nitroprusside (Supplemental table S2). During the 3 months follow-up, three patients missed fewer than 10 doses of empagliflozin, two missed between 10 and 30 doses, two missed between 31 and 50 doses, and four missed more than 50 doses.

### General cardiometabolic effects of empagliflozin

During the treatment period with empagliflozin, no significant changes were observed in body mass index, blood pressure, fasting triglycerides, fasting insulin, C-reactive protein and free thiols, a marker of systemic oxidative stress (Table 2). In the per-protocol group (*n* = 26), 19 patient had no medication changes. Diuretic doses were increased in 2 patients (8%), decreased in 2 (8%) and discontinued in 3 (12%).

### Endothelin-1 in plasma

**Fig. 4:**
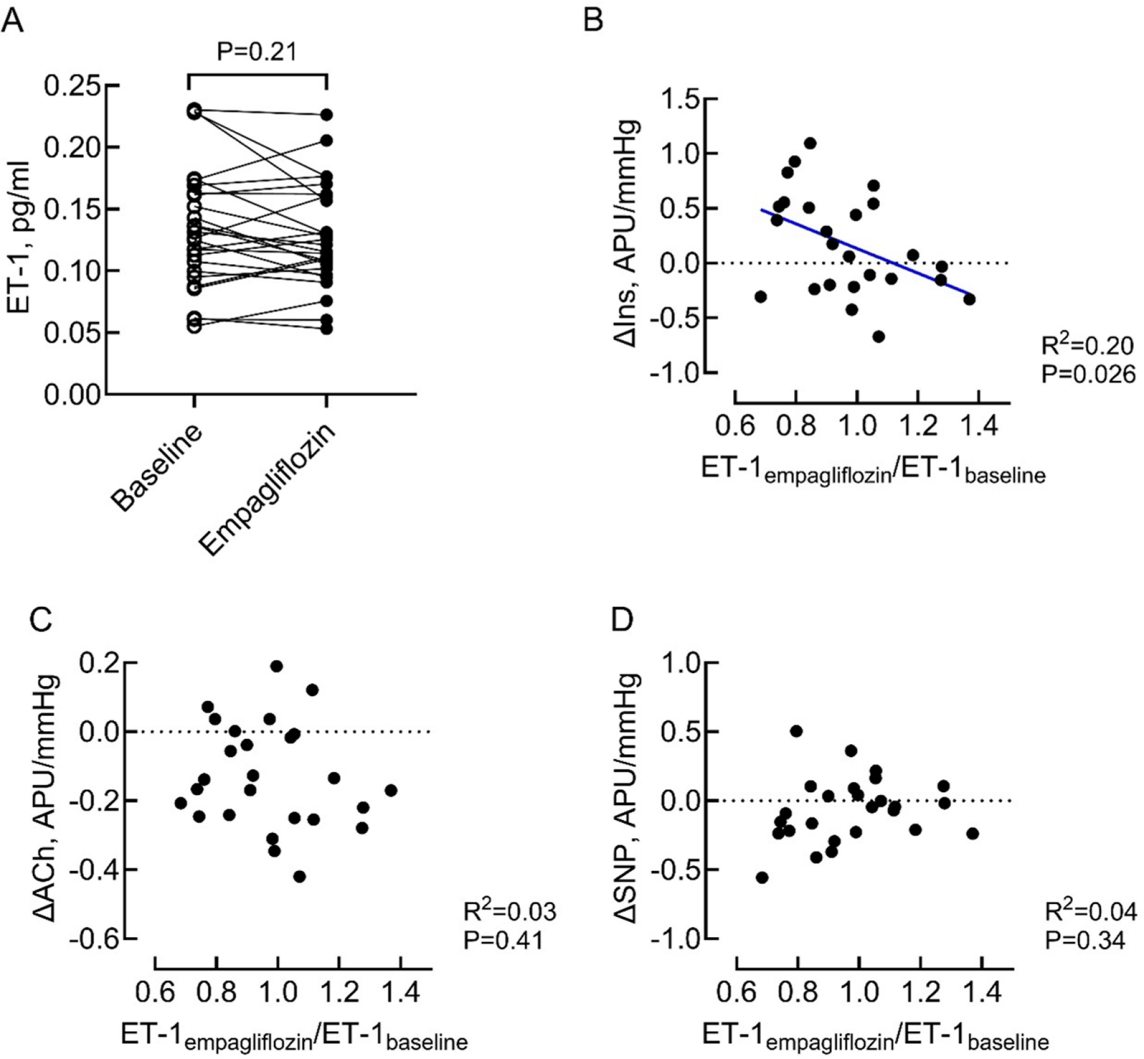
Endothelin levels before and after empagliflozin treatment in HFpEF patients. **A** Endothelin levels in plasma before (baseline) and after 3 months of empagliflozin treatment. **B–D** Correlations between changes in plasma ET-1 during empagliflozin treatment and skin blood flow stimulated by insulin (**B**), acetylcholine (**C**) and sodium nitroprusside (**D**). *CVC* Cutaneous vascular conductance

Treatment with empagliflozin did not affect plasma concentrations of ET-1 compared to baseline (1.1 pg/ml [1.0–1.4] after treatment vs. 1.3 pg/ml [1.1–1.6] at baseline) *P* = 0.21; Fig. 4A). After 3 months of follow-up, 16/25 participants showed a decrease in plasma ET-1 during empagliflozin treatment. In the intention to treat analysis, the effect of empagliflozin on plasma ET-1 was also not significant (*P* = 0.096). Insulin-stimulated skin blood flow after empagliflozin treatment weakly associated with the change in plasma ET-1 concentration during empagliflozin treatment (*R* = 0.44, *p* = 0.026; Fig. 4B). Responses to acetylcholine (Fig. 4C) and SNP (Fig. 4D) did not relate to the change in plasma ET-1 during empagliflozin treatment.

**Fig. 5:**
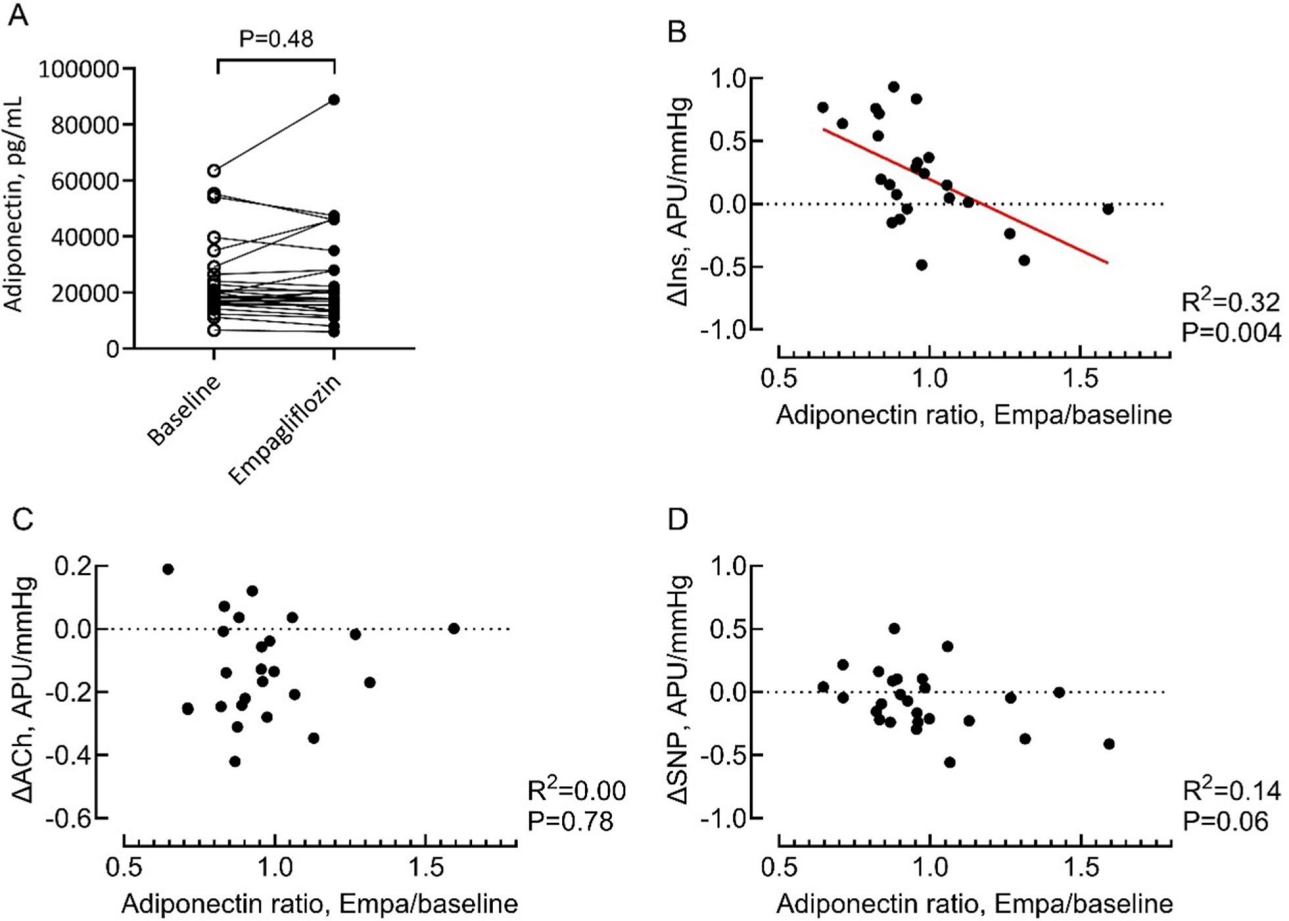
Adiponectin levels before and after empagliflozin treatment in HFpEF patients. **A** Adiponectin levels in plasma before (baseline) and after 3 months of empagliflozin treatment. **B–D** Correlations between changes in plasma ET-1 during empagliflozin treatment and skin blood flow stimulated by insulin (**B**), acetylcholine (**C**) and sodium nitroprusside (**D**). *CVC* Cutaneous vascular conductance

As adipose tissue determines microvascular responses to insulin and adiponectin is an important adipokine mediating this interaction, we studied whether adiponectin in plasma relates to the effects of empagliflozin on microvascular function in HFpEF. Similarly to ET-1, we did not observe a systemic change in adiponectin levels during empagliflozin treatment (Fig. 5A), but a decrease in plasma adiponectin levels specifically associated with increased microvascular flow responses to insulin (Fig. 5B–D). No relationship with ACh or SNP responses was observed.

### Quality of life

**Fig. 6:**
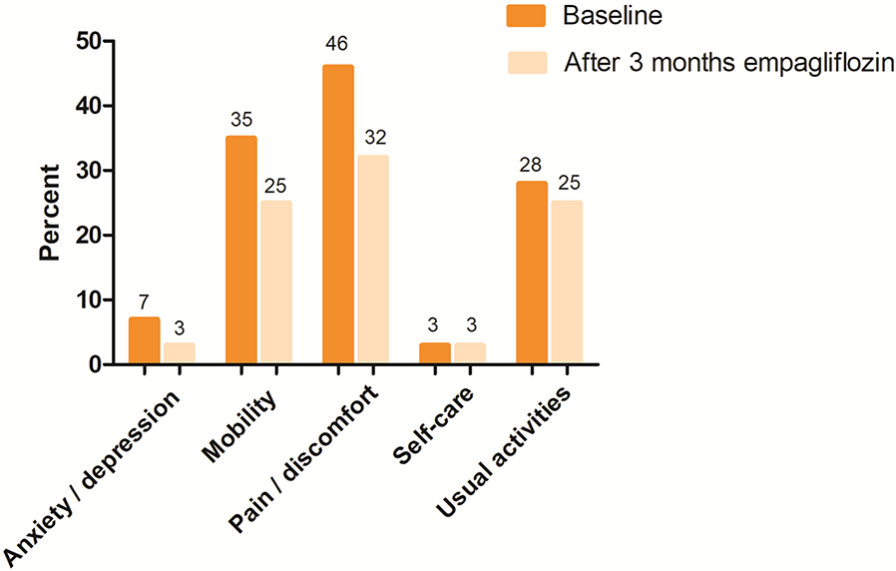
Patients reporting moderate or severe problems of EQ-5D-5 L dimensions at baseline and 3 months follow-up

In the per-protocol analysis (*n* = 28), the PCHC results showed 12 participants (42.9%) with overall improvement in quality of life, six participants (21.4%) with overall worsening, six participants (21.4%) showed a mixed change, two participants (7.1%) showed no change, and two participants (7.1%) showed no problems during both visits (Table 4). At 3 months follow-up compared to baseline, patients reported fewer moderate or severe problems in all EQ-5D dimensions, most prominently in the dimensions of mobility and pain/discomfort (Fig. 6, supplemental table S3). The mean levels of the EQ-5D-5 L index and the VAS score did not significantly change between baseline and after three months. **(**Table 5**).** No significant correlations were found between the CVC and the EQ-5D-5 L index or VAS score.

**Table 4:** Health changes after 3 months of empagliflozin according to the Paretian classification of health change (PCHC)

PCHC	Per protocol (*n* = 28)		Intention to treat (*n* = 39)	
	N	%	N	%
Improved	15	38.5	12	42.9
Mixed change	8	20.5	6	21.4
No change	5	12.8	2	7.1
No problems	2	5.1	2	7.1
Worsen	9	23.1	6	21.4

Patients who reported the same classification of health at baseline and 3 months follow-up were represented as ‘no change’. Patients who never reported any health problems were represented as ‘no problems’. Patients who did not consistently improve or worsen on all aspects of the classification were represented as ‘Mixed change’

**Table 5:** Mean levels of EQ-5D index and the visual analogue scale (VAS) by visit

	Baseline	Follow-up at 3 months	*P*-value
	Mean ± SD	Mean ± SD	
*Intention to treat*			
EQ VAS	69 ± 14	72 ± 13	0.364
EQ-5D index	0.78 ± 0.14	0.79 ± 0.17	0.714
*Per protocol*			
EQ VAS	69 ± 16	75 ± 15	0.219
EQ-5D index	0.78 ± 0.14	0.80 ± 0.19	0.411

## Discussion

To our knowledge, this study is the first to report on the effects of the SGLT-2 inhibitor empagliflozin on peripheral microvascular function in response to endothelium-dependent and independent stimuli in patients with HFpEF. Our findings reveal an association between three months of empagliflozin treatment in HFpEF and changes in endothelium-dependent microvascular function. We observed an improved endothelium-dependent blood flow response to insulin measured by LASCA in the forearm skin. This effect of empagliflozin was not accompanied by a generalised improvement of endothelium-dependent vasodilation, as we observed decreased responses to acetylcholine, nor did we observe endothelium independent changes, assessed by response to sodium nitroprusside.

### The role of insulin in endothelial function and the interplay with SGLT2-inhibitors

A prior study demonstrated an improved vascular response (vascular stiffness and microvascular perfusion) to insulin in cardiac and skeletal muscles in 11 subjects with type II diabetes after treatment with empagliflozin [43]. Despite the low number of patients with diabetes mellitus in our study population, it is likely that insulin resistance is present to some extent in our multi-morbid HFpEF population. These patients often suffer from a combination of comorbidities such as hypertension and obesity, leading to increased oxidative stress and inflammation [44, 45].

In contrast, the EMBLEM study, a multi-centre placebo-controlled double-blind randomized trial of 105 subjects with type 2 diabetes and cardiovascular disease, did not identify any positive or negative effect on peripheral microvascular function after a 24-week treatment with empagliflozin [46]. The latter study assessed reactive hyperaemia index (RHI) as measured by peripheral arterial tonometry (EndoPAT). This measurement reflects a combination of endothelial function as well as endothelium—independent mechanisms [47]. Similarly, RHI did not improve after exercise training in HFpEF [48]. The combination of mechanisms assessed using RHI could have led to these neutral results, while our study assessed endothelium-dependent changes specifically. This underscores the complexity of assessing microvascular and endothelial (dys)function.

The combination of an improved vasodilator response to insulin and diminished response to acetylcholine observed in our study can be explained by a selective effect of empagliflozin on insulin-mediated responses, and different mediators of microvascular responses to insulin and acetylcholine. Insulin affects the vascular endothelium via various pathways, resulting in either vasodilation or vasoconstriction. In healthy conditions, insulin stimulates nitric oxide (NO) production and relaxation of vascular smooth muscle cells via phosphatidylinositol 3-kinase (Pl3K) and Akt-mediated phosphorylation of endothelial nitric oxide synthase (eNOS) [36]. In inflammatory conditions such as type 2 diabetes, insulin can increase endothelin-1 (ET-1) leading to vasoconstriction via the mitogen-activated protein kinase (MAPK) pathway. This imbalance can be reversed by SGLT2 inhibition [49, 50], as empagliflozin increases levels of the adipokine adiponectin which reduces ET-1 activity and specifically enhances insulin-induced vasodilatation [51, 52]. In HFpEF, overproduction of ET-1 is a feature of endothelial dysfunction and related to an increase of symptoms resulting in hospitalisations [53]. Unlike insulin, vasodilator effects of acetylcholine in precapillary arterioles are partially mediated by hydrogen peroxide. Empagliflozin reduces H2O2 production in HFpEF [25], potentially explaining the decrease in acetylcholine responses we observed.

Importantly, the improvement in insulin-stimulated blood flow in the skin during empagliflozin treatment appears largely independent of changes in systemic ET-1 levels, indicating that mechanisms beyond ET-1 reduction contribute to empagliflozin’s effects on microvascular function during hyperinsulinemia. Improved insulin sensitivity may be a potential contributing factor to the insulin specific effects of empagliflozin observed in our study. Although oxidative stress plays a major role in the mechanisms of insulin resistance, and empagliflozin appears to mitigate oxidative stress, this is unlikely the primary mechanism for improved insulin sensitivity in HFpEF [24, 54–56]. As such effect would also be expected to improve acetylcholine dependent vasodilation, and we observed a decreased vasodilation in the present study.

Another potential mechanism affecting insulin specific effects of SGLT-2 inhibition involves adipose tissue. SGLT-2 inhibitors have been shown to reduce epicardial adipose tissue, which causes a decrease secretion of pro-inflammatory adipokines such as Il-6, and increase adiponectin levels [57, 58]. Adiponectin increases AMP-activated protein kinase (AMPK) activity, which is thought to enhance insulin sensitivity in peripheral tissues [52, 59, 60]. Besides this indirect effect, empagliflozin may also directly activate AMPK in the endothelium [61, 62]. Surprisingly, we observed a paradoxical inverse relationship between effects of empagliflozin on plasma adiponectin levels and insulin-stimulated blood flow (see graphical abstract). This “adiponectin paradox” is in line with the finding that high adiponectin predicts heart failure [63]. In this light, it should be noted that adiponectin is an outside-in messenger produced by perivascular fat tissue, binding to vascular AdipoR1/R2 receptors. Plasma levels of adiponectin are therefore determined by production as well as adiponectin receptor expression in the vessel wall. Expression of AdipoR1/R2 is decreased in experimental HFpEF [64], and this decrease in receptors can increase plasma levels of adiponectin while decreasing its bioactivity. The exact role of vascular adiponectin signaling in HFpEF remains to be determined.

Even though the cardiovascular benefits of empagliflozin in HFpEF have been shown in patients with and without diabetes mellitus [12], the results of our study raise the question whether patients with insulin resistance benefit more from empagliflozin treatment in terms of microvascular function. Further research is warranted to investigate this hypothesis.

### Acetylcholine and sodium nitroprusside: empagliflozin affects specific pathways in microvascular function

The current results suggest that empagliflozin does not have a generalised effect on peripheral microvascular vasodilator function in HFpEF. Generalised effects on endothelial and smooth muscle cells would have improve responses to both acetylcholine and nitroprusside. Instead, we observed decreased acetylcholine and stable nitroprusside responses, suggesting that the effects on microvascular function by empagliflozin are insulin specific, without direct impact on smooth muscle cells.

Acetylcholine induces vasodilation through three primary pathways. Firstly, it increases NO production via activating the muscarinic M3 receptor on endothelial cells. Secondly, acetylcholine activates the cyclooxygenase (COX) pathway, generating prostacyclin (PGI2), to increase cyclic adenosine monophosphate (cAMP) and hyperpolarise the smooth muscle cell. The third potential pathway is via endothelial-derived hyperpolarizing factors (EDHF). The EDHF pathway, particularly the EDHF hydrogen peroxide (H2O2), is predominant in the microcirculation [37, 65]. Furthermore, in a state of diminished NO bioavailability, compensatory upregulation of EDHF may occur [66]. In the myocardium of patients with HFpEF empagliflozin has been shown to reduce H2O2 by reducing reactive oxygen species [25]. As H2O2 is a mediator of endothelium-dependent vasodilatation in human arterioles, functioning as an EDHF, this may explain the reduction of acetylcholine responses we observed [51, 67].

Combining the previous results with our new data, we propose that empagliflozin treatment in HFpEF influences vasodilator homeostasis in the microcirculation, with specific insulin mediated effects.

### Clinical implications and future directions

The current findings suggest that empagliflozin may have specific effects on microvascular endothelial function in HFpEF. This observation paves the way for further research to investigate whether specific endothelial-specific pathways relate to improved outcomes in HFpEF, which can result in more specific treatments targeting endothelial dysfunction. Furthermore, if we identify patients based on their microvascular function profile, this may lead to possibilities for personalised treatment approaches. The observed differential effects on insulin and acetylcholine responses highlight the complex pathways in the microvasculature, which remain to be further elucidated in relation to HFpEF pathophysiology. The opposing effects we found for insulin, acetylcholine and sodium nitroprusside underscore the importance of comprehensive endothelial function assessment in evaluating novel therapies for HFpEF. This study adds to the existing evidence of the effect of empagliflozin in HFpEF and was the first to show that peripheral microvascular endothelial function is a druggable target in these patients.

### Study limitations

Certain limitations should be considered when interpreting the current study results. Firstly, the absence of a control or placebo group limits our ability to definitely attribute the observed microvascular effects to the empagliflozin treatment, rather than other confounding factors. However, while our patients who prematurely stopped the treatment had similar baseline characteristics, the microvascular effect was absent without full 3 months of empagliflozin use, which strengthens the likeliness that the observed effects are a treatment effect. Secondly, while LASCA is known for its reproducibility and was performed under controlled circumstances, reference values in a healthy population and effects of potential day to day variations in microvascular function are presently lacking. Therefore, our analysis is limited to comparisons between baseline and follow-up, but we could not determine the absolute presence or severity of microvascular dysfunction. Lastly, the small sample size and specific Caucasian patient cohort, included from a single centre specialised HFpEF outpatient clinic, may limit the statistical power and external validity of our results.

## Conclusions

This study shows that three months of empagliflozin treatment changed peripheral microvascular function in patients with HFpEF. Our results suggest that empagliflozin enhances microvascular blood flow, as measured by LASCA, specifically via vascular actions of insulin, rather than a general effect on endothelial- or smooth muscle cell function. Moreover, this study proves that microvascular function is a modifiable target in HFpEF. Novel treatment approaches in HFpEF may be found through further research on the underlying modifiable microvascular mechanisms in HFpEF.

## Electronic supplementary material

Below is the link to the electronic supplementary material.

Supplementary Material 1.

## Data Availability

The datasets used and/or analysed during the current study are available from the corresponding author on reasonable request.

## Abbreviations

Ach: Acetylcholine
AMPK: AMP-activated protein kinase
APU: Arbitrary perfusion units
AUC: Area under the curve
cAMP: Cyclic adenosine monophosphate
cGMP: Guanosine 3′,5′-cyclic monophosphate
COX: Cyclooxygenase
CVC: Cutaneous vascular conductance
EDHF: Endothelial derived hyperpolarizing factors
eNOS: Endothelial nitric oxide synthase
ET-1: Endothelin-1
HFpEF: Heart failure with preserved ejection fraction
INS: Insulin
IP: Prostaglandin I2 receptor
K +: Potassium ion
LASCA: Laser speckle contrast analysis
MAPK: Mitogen-activated protein kinase
M3: Muscarinic receptor 3
MO: Mobility
MVD: Microvascular dysfunction
NO: Nitric oxide
PCHC: Paretian Classification of Health Change
PGI2: Prostacyclin
Pl3K: Phosphatidylinositol 3-kinase
RHI: Reactive hyperaemia index
ROS: Reactive oxygen species
SNP: Sodium nitroprusside
