# Supplementary material for: The effect of empagliflozin on peripheral microvascular dysfunction in patients with heart failure with preserved ejection fraction
Source: Cardiovasc Diabetol. 2025 Apr 25;24:182. doi: 10.1186/s12933-025-02679-8 (PMC12023568; doi:10.1186/s12933-025-02679-8)
Supplement: Supplementary file 1 — Supplementary Material 1. [file 12933_2025_2679_MOESM1_ESM.doc]

**Supplementary material**

**The effect of empagliflozin on peripheral microvascular dysfunction in heart failure with preserved ejection fraction**

**Address for correspondence**Vanessa van Empel

Maastricht University Medical Centre+, PO Box 616, 6200 MD Maastricht, The Netherlands

Fax: +31 43 387 5104
Telephone: +31 43 387 7095
E-mail: [vanessa.van.empel@mumc.nl](mailto:vanessa.van.empel@mumc.nl)

**Appendix 1: Protocol for laser speckle contrast analysis (LASCA) with iontophoresis of acetylcholine (Ach), insulin (INS), and sodium nitroprusside (SNP)**

# Measures for reproducibility

- Check, regulate, and record room temperature.
- Warm the patient's hands if they are cold.
- The patients must be present in the room for 20 minutes and has not performed physical activity prior to the measurements
- The patient must be fasting during the measurement (Medication use as usual is allowed)
- Position the patient comfortably with arm support, preferably in supine position in bed.
- Instruct the patient to remain still and silent during the measurement.
- Conduct follow-up measurements on the same arm as baseline.

**Preparation for iontophoresis**

- Clean the ventral side of the forearm with alcohol. Use the dominant arm for insulin, and the non-dominant arm for acetylcholine and sodium nitroprusside.
- Place the drug delivery electrodes on the ventral side of the arm below the elbow, avoiding large veins, hairy areas, and other skin irregularities.
- Place the dispersive electrodes approximately 10 cm distal to the drug delivery electrodes
- Inject 0.4ml of one of the selected vasoactive substance into the drug delivery electrodes, minimizing air bubbles.
  - Acetylcholine (Miochol-E) 1%
  - Sodium nitroprusside 0.1%
  - Insulin (novorapid) 100 IU/ml
- Connect the PeriIont wires to their respective poles on both the dispersive and drug delivery electrode, ensuring that the wires do not overlap.

**Preparation for LASCA**

- Start Pimsoft software
- Position LASCA camera between 10-40 cm distance of the arm
- Choose the "averaging" function, this was set at 40x.
- Make one region of interest (ROI) in each drug delivery electrode
- Start the measurement right before starting the iontophoresis protocol

**Iontophoresis protocol**

*Acetylcholine*

- Dose acetylcholine: 20 seconds 100 µA (0.10 mA) / 40 seconds rest (x7).
- Drug polarity positive setting.
- 0.00-1.00 min baseline
- 1.00-1.20 min 1st dose Ach
- 2.00-2.20 min 2nd dose Ach
- 3.00-3.20 min 3rd dose Ach
- 4.00-4.20 min 4th dose Ach
- 5.00-5.20 min 5th dose Ach
- 6.00-6.20 min 6th dose Ach
- 7.00-7.20 min 7th dose Ach
- 8.00 min stop protocol
- Save/back-up measurement

*Sodium nitroprusside*

- Dose SNP: 20 seconds 200 µA (0.20 mA) / 70 seconds rest (x9)
- Drug polarity negative setting.
- 0.00-1.00 min baseline
- 1.00-1.20 min 1st dose SNP
- 2.30-2.50 min 2nd dose SNP
- 4.00-4.20 min 3rd dose SNP
- 5.30-5.50 min 4th dose SNP
- 7.00-7.20 min 5th dose SNP
- 8.30-8.50 min 6th dose SNP
- 10.00-10.20 min 7th dose SNP
- 11.30-11.50 min 8th dose SNP
- 13.00-13.20 min 9th dose SNP
- 14.30 min stop protocol
- Save/ back-up measurement

*Insulin*

- Insulin dose: 18 minutes 20 µA (0.02 mA), continuous.
- Drug polarity negative setting.
- 0.00-1.00 min baseline
- 1.00-19.00 min 1st dose (20 µA)
- 19.00 min Stop protocol
- Save/ back-up measurement

**Appendix 2: Supplemental tables**

**Table S1. Predictors for difference in blood flow response between baseline and 3 months follow up**

|  | **Unadjusted** | | | **Adjusted *** | | |
| --- | --- | --- | --- | --- | --- | --- |
|  | **Bèta (95% CI)** | **Standandardized beta** | **p-value** | **Bèta (95% CI)** | **Standandardized beta** | **p-value** |
| **Sodium nitroprusside** |  |  |  |  |  |  |
| Creatinine | 0.006 (0.002 to 0.010) | 0.533 | 0.005 | **0.007 (0.002 to 0.011)** | **0.604** | **0.007** |

*Adjusted for age, sex, and New York Heart Association class

**Table S2. Intention to treat analysis: blood perfusion as measured by laser speckle constrast analysis during iontophoresis of acetylcholine (N=37)**

|  | **Baseline** | **Follow-up at 3 months** | P-value |
| --- | --- | --- | --- |
| **Acetylcholine (n = 37)** |  |  |  |
| Baseline perfusion (APU) | 47 [40 – 68] | 41 [34 – 49] | 0.008 |
| Baseline perfusion (APU/mmHg)* | 0.49 ± 0.14 | 0.45 ± 0.13 | 0.089 |
| Peak perfusion (APU) | 123 ± 30 | 108 ± 22 | 0.002 |
| Peak perfusion (APU/mmHg)* | 1.22 ± 0.29 | 1.11 ± 0.27 | 0.023 |
| Absolute change (APU) | 74 ± 27 | 65 ± 19 | 0.012 |
| CVC (APU/mmHg)* | 0.73 ± 0.25 | 0.66 ± 0.20 | 0.059 |
| Area under the curve | 21274 ± 8686 | 19116 ± 8957 | 0.158 |
| **Insulin (n=35)** |  |  |  |
| Baseline perfusion (APU) | 56 [44- 64] | 44 [36 – 50] | < 0.001 |
| Baseline perfusion (APU/mmHg)* | 0.55 [0.45 – 0.64] | 0.48 [0.36 – 0.52] | 0.001 |
| Peak perfusion (APU) | 126 ± 29 | 108 ± 22 | 0.006 |
| Peak perfusion (APU/mmHg)* | 1.26 ± 0.33 | 1.11 ± 0.23 | 0.022 |
| Absolute change (APU) | 68 ± 24 | 65 ± 22 | 0.514 |
| CVC (APU/mmHg)* | 0.68 ± 0.24 | 0.66 ± 0.21 | 0.659 |
| Area under the curve | 31836 [23995 – 43151] | 32055 [22903 – 40566] | 0.331 |
| **Sodium nitroprusside (n=37)** |  |  |  |
| Baseline perfusion (APU) | 44 ± 11 | 41 ± 12 | 0.178 |
| Baseline perfusion (APU/mmHg)* | 0.44 ± 0.13 | 0.43 ± 0.14 | 0.607 |
| Peak perfusion (APU) | 105 ± 43 | 112 ± 35 | 0.331 |
| Peak perfusion (APU/mmHg)* | 1.05 ± 0.46 | 1.18 ± 0.37 | 0.113 |
| Absolute change (APU) | 62 [18 – 103] | 70 [51 – 99] | 0.202 |
| CVC (APU/mmHg)* | 0.64 [0.18 – 0.94] | 0.68 [0.55 – 0.96] | 0.163 |
| Area under the curve | 32400 [7365 – 67592] | 43469 [27230 – 64466] | 0.122 |

Legend: Data presented as median [interquartile range], or mean ± standard deviation, as appropriate. APU, arbitrary perfusion units. CVC, cutaneous vascular conductance (Absolute change / mean arterial pressure). *perfusion / mean arterial pressure

**Table S3. Patient distribution of categorical responses across EQ-5D-5L dimensions by intention-to-treat and per-protocol analyses.** Each dimension is rated on a 5-point scale (1–5), i.e. no problem, slight problem, moderate problem, severe problem, and extreme problem.

| **EQ-5D dimension** | | **Baseline** | | | | | | **3- months Follow-up** | | | |
| --- | --- | --- | --- | --- | --- | --- | --- | --- | --- | --- | --- |
|  |  | Intention to treat (n = 39) | | | Per protocol  (n = 28) | | | Intention to treat (n = 39) | | Per protocol  (n = 28) | |
|  |  | **N** | **(%)** | **N** | | **(%)** | **N** | | **(%)** | **N** | **(%)** |
| Mobility | 1 | 11 | (28.2) | 7 | | (25.0) | 12 | | (30.8) | 9 | (32.1) |
|  | 2 | 15 | (38.5) | 11 | | (39.3) | 16 | | (41.0) | 12 | (42.9) |
|  | 3 | 9 | (23.1) | 8 | | (28.6) | 6 | | (15.4) | 5 | (17.9) |
|  | 4 | 4 | (10.3) | 2 | | (7.1) | 5 | | (12.8) | 2 | (7.1) |
|  | 5 | 0 | (0) | 0 | | (0) | 0 | | (0) | 0 | (0) |
| Self-care | 1 | 32 | (82.1) | 23 | | (82.1) | 35 | | (89.7) | 25 | (89.3) |
|  | 2 | 6 | (15.4) | 4 | | (14.3) | 3 | | (7.7) | 2 | (7.1) |
|  | 3 | 1 | (2.6) | 1 | | (3.6) | 0 | | (0) | 0 | (0) |
|  | 4 | 0 | (0) | 0 | | (0) | 1 | | (2.6) | 1 | (3.6) |
|  | 5 | 0 | (0) | 0 | | (0) | 0 | | (0) | 0 | (0) |
| Usual activities | 1 | 15 | (38) | 8 | | (28.6) | 17 | | (43.6) | 13 | (46. 4) |
|  | 2 | 13 | (33.3) | 12 | | (42.9) | 12 | | (30.8) | 8 | (28.6) |
|  | 3 | 10 | (25.6) | 7 | | (25) | 8 | | (20.5) | 5 | (17.9) |
|  | 4 | 1 | (2.6) | 1 | | (3.6) | 2 | | (5.1) | 2 | (7.1) |
|  | 5 | 0 | (0) | 0 | | (0) | 0 | | (0) | 0 | (0) |
| Pain and discomfort | 1 | 12 | (30.8) | 9 | | (32.1) | 15 | | (38.5) | 13 | (46.4) |
|  | 2 | 13 | (33.3) | 6 | | (21.4) | 13 | | (33.3) | 6 | (21.4) |
|  | 3 | 13 | (33.3) | 12 | | (42.9) | 10 | | (25.6) | 8 | (28.6) |
|  | 4 | 1 | (2.6) | 1 | | (3.6) | 1 | | (2.6) | 1 | (3.6) |
|  | 5 | 0 | (0) | 0 | | (0) | 0 | | (0) | 0 | (0) |
| Anxiety and depression | 1 | 31 | (79.5) | 22 | | (78.6) | 29 | | (74.4) | 20 | (71.4) |
|  | 2 | 6 | (15.4) | 4 | | (14.3) | 9 | | (23.1) | 7 | (25) |
|  | 3 | 2 | (5.1) | 2 | | (7.1) | 1 | | (2.6) | 1 | (3.6) |
|  | 4 | 0 | (0) | 0 | | (0) | 0 | | (0) | 0 | (0) |
|  | 5 | 0 | (0) | 0 | | (0) | 0 | | (0) | 0 | (0) |
